# It's Daddy's Turn! Development of Turn‐Taking in Infant‐Father Interactions and Associations With Paternal Mentalizing and Involvement

**DOI:** 10.1111/infa.70115

**Published:** 2026-08-10

**Authors:** Selina Ismair, Antonia Dinzinger, Beate Priewasser, Karl Heinz Brisch, Gabriela Markova

**Affiliations:** ^1^ Institute for Early Life Care Paracelsus Medical Private University Salzburg Austria; ^2^ Department of Pediatrics University Hospital Salzburg Paracelsus Medical Private University Salzburg Austria; ^3^ Dr. von Hauner Children's Hospital Ludwig Maximilians University Munich Germany

**Keywords:** communicative development, father involvement, father‐infant interaction, mentalizing, protoconversations, turn‐taking

## Abstract

Turn‐taking, the coordinated exchange of communicative signals between infants and caregivers, is vital for child communicative development. While it is considered a human universal, most research has focused on mother‐infant dyads. Given fathers' increasing caregiving involvement, understanding their contribution to early communication is essential. Thus, we have examined early infant‐father turn‐taking and the role of fathers' mentalizing abilities, which may support turn prediction, and paternal involvement, as it may shape the frequency and quality of exchanges. We observed 39 father‐infant dyads in natural interactions at 7 weeks and 6 months postpartum. Vocalizations were micro‐coded and turn‐taking was defined as a vocal response by one partner within 3000 ms of the other's vocalization. Mentalizing was measured via the Reflective Functioning Scale of the Parent Development Interview, while involvement was indexed by weeks of paternity leave. Results showed little change in turn‐taking over time, except for a slight decline in infant turns and increase in paternal overlaps. Mixed effect models revealed significant between‐dyad variability; higher mentalizing was associated with fewer paternal overlaps, and greater involvement was linked to shorter overlap durations. Findings suggest that infants are active turn‐takers from early on and paternal mentalizing may support attuned coordination. Despite a relatively homogenous sample, results underscore fathers' importance in early communicative development.

## 1 Introduction

Interactions with caregivers are crucial for infant survival, fulfilling physiological, emotional, and attachment needs, thereby shaping infants' learning environment (e.g., Parsons et al. 2010; Tamis‐LeMonda and Masek 2023). A key component of early interactions is the turn‐taking communication between infants and caregivers, that is the rapid and reciprocal alternation of vocalizations (Cosper and Pika 2025; Levinson 2016). These early turn‐taking exchanges are predictive of child linguistic, cognitive and socio‐emotional outcomes (e.g., Gilkerson et al. 2018; Gómez and Strasser 2021; Zhang et al. 2024). However, research on the development and predictors of vocal turn‐taking remains limited, with most studies focusing on mother‐infant dyads, leaving interactions between infants and their fathers largely unexplored. Therefore, the overall aim of the present study was to systematically investigate vocal turn‐taking dynamics in father‐infant dyads.

Infants engage in social communication long before they can speak, using proto‐conversational sounds—known as protophones—that serve as precursors to verbalizations (Kent 2022). Caregivers are highly responsive to these sounds, treating them as speech‐like acts and responding in a well‐timed, coordinated manner that mirrors the structure of adult conversations (Bateson 1975; Yoo et al. 2018). A defining characteristic of these communicative exchanges is their turn‐taking pattern, that is the reciprocal alternation of utterances between interlocutors, including minimal overlap and brief response times, which together create a smooth conversational flow (Stivers et al. 2009). Interestingly, parents can distinguish infant protophones from other vocalizations, such as crying, and selectively engage in turn‐taking with these speech‐like sounds while tending to overlap with cries, as their primary goal in such instances is co‐regulation rather than communication (Yoo et al. 2018). By responding to infants' protophones from very early in life, parents provide infants with opportunities to experience the temporal structure of interpersonal conversations, which has been suggested to play a crucial role in the development of timing skills and turn‐taking (Cosper and Pika 2025).

While turn‐taking can be observed in different modalities (e.g., Rohlfing et al. 2020), research indicates that vocal turn‐taking is associated with interbrain synchronization (e.g., Lin et al. 2023), regulation of stressful interactions (Weisman et al. 2016) and the development of attachment and language (e.g., Ginsburg and Kilbourne 1988; Jaffe et al. 2001; Klann‐Delius and Hofmeister 1997). For example, recent studies showed a relationship between conversational turn‐taking frequency in infancy and children's vocabulary at 2 years of age (Donnelly and Kidd 2021; V. Nguyen et al. 2022). Importantly, it is not the mere exposure to language itself that matters most, but the reciprocal act between adult and child that plays a particular role in children's language development (Donnelly and Kidd 2021; Lopez et al. 2020; Zhang et al. 2024; Zimmerman et al. 2009). By examining turn‐taking as a fundamental aspect of early conversations, we can gain a deeper understanding of the development of human communication.

It has been argued that infants are equipped from birth with an infrastructure for social interactions that forms the foundation for vocal turn‐taking (Levinson 2016). Supporting this claim, several studies have demonstrated that turn‐taking begins early in infancy, with communicative turn‐taking structures observed in interactions with infants as young as 6–12 weeks (Crown et al. 2002; Gratier et al. 2015; Hilbrink et al. 2015). Some findings suggest that turn‐taking may even emerge soon after delivery, because infants are capable of perceiving contingencies immediately after birth and develop expectations for behavioral events (e.g., Nagy and Molnar 2004). Indeed, studies of 2‐4‐day‐old newborns revealed that infants are active participants in face‐to‐face interactions with their caregivers, as their vocalizations rarely occur in isolation and caregivers respond to them swiftly, with pauses often lasting less than a second between turns (Boiteau et al. 2021; Dominguez et al. 2016). These rapid exchanges exemplify the mutual engagement that defines early turn‐taking and highlight the sophistication of early communicative exchanges (Boiteau et al. 2021; Dominguez et al. 2016).

As children mature, turn‐taking evolves, with interactional patterns becoming increasingly complex and pronounced. The initial goal is to avoid overlaps when exchanging turns, while optimizing response latency (V. Nguyen et al. 2022). However, overlapping vocalizations are not necessarily a sign of poor attunement, but may also reflect normative developmental characteristics of early communication (Jeong and Ha 2025). For example, Ginsburg and Kilbourne (1988) observed that the youngest infants in their sample of 7‐18‐weeks‐old exhibited more overlapping vocalizations than alternating vocalizations. Between 12‐ and 18‐weeks, infants and caregivers increasingly alternate vocalizations (e.g., Beebe et al. 1988; Ginsburg and Kilbourne 1988), marking the emergence of a rudimentary turn‐taking structure resembling adult conversational exchange. Developmental changes in response latency appear less linear. While some studies suggest that optimal response latencies increase as infants begin producing more linguistically complex vocalizations (e.g., Hilbrink et al. 2015; Jeong and Ha 2025; Levinson 2016), they may decrease again as communicative efficiency improves with language acquisition (Lindsay et al. 2019; V. Nguyen et al. 2022). Yet, the precise timing of these developmental shifts remains unclear (see Cosper and Pika 2025, for a recent review), with evidence pointing to individual and context variability (Casillas et al. 2016; Gratier et al. 2015; Hilbrink et al. 2015), highlighting the need for further longitudinal research.

While studies on turn‐taking provide valuable insights into early communicative development, there are still many unanswered questions. Most importantly, since previous research has focused almost exclusively on mother‐infant dyads, there is a pressing need for more representative data on turn‐taking including different social contexts and interaction partners (Dominguez et al. 2016; Meunier et al. 2024; V. Nguyen et al. 2022). This highlights the potential value of studying fathers to better understand the development of turn‐taking with different interaction partners and to gain a more comprehensive view of the communicative environment surrounding infants. Interestingly, while a recent review on turn‐taking development highlights the lack of cultural diversity in existing studies (Cosper and Pika 2025), it does not mention the equally striking absence of fathers as interaction partners. Although fathers are spending more time with their infants from the earliest stages of life (e.g., Altenburger and Schoppe‐Sullivan 2020; Bakermans‐Kranenburg et al. 2019), investigations of father‐infant interactions are rare and existing studies show both similarities and differences between infant social exchanges with mothers and fathers (e.g., Kelly et al. 2022).

Evidence for similar turn‐taking structures in mothers and fathers comes from a vocal imitation study showing that during interactions with their 2–6‐month‐old infants', both caregivers display comparable amount and duration of vocal imitation, turn‐taking and co‐action (i.e., vocal overlap; Kokkinaki and Vasdekis 2003). Additionally, both neonates and fathers were found to demonstrate initiative and timely engagement in turn‐taking (Boiteau et al. 2021). These findings indicate infants' early intention for social exchange with their fathers and highlight fathers' sensitivity to infant cues. In contrast, there is evidence of potential differences in turn‐taking between parents. For instance, fathers were found to provide less vocal input than mothers (Johnson et al. 2014; Kiepura et al. 2022), and this difference may further influence their turn‐taking patterns. In fact, fathers were found to engage in shorter and less frequent turn‐taking sequences and elicit less responsiveness from their neonates (Boiteau et al. 2021; Dominguez et al. 2016). Given these mixed findings, it is critical to expand research on infant‐father vocal turn‐taking. Specifically, existing studies are limited by their cross‐sectional designs and focus on a very narrow age range. Consequently, systematic longitudinal investigations of vocal turn‐taking behavior between infants and fathers are needed to provide deeper insights and address the unresolved question posed by Bureau et al. (2014) whether father–child interactions involve distinct dynamics or reflect similar mechanisms as those observed in mother–child relationships.

In fact, the factors influencing the development of turn‐taking structures in parent‐child interactions, as well as the specific mechanisms underlying this phenomenon, are still not fully understood (Cosper and Pika 2025). Closely coordinated turn‐taking exchanges require the ability to recognize and predict the communicative intentions of the other person (e.g., anticipating the end of a turn; Levinson 2016; Templeton et al. 2022), creating a sense of connection between the communicators. One construct that may contribute to this process is mentalizing, defined as the ability to reflect on one's own and others' mental states (Fonagy and Allison 2012). Mentalizing may enable caregivers to infer the intentions underlying their children's behavior more accurately (see Buttitta et al. 2019) and is often expressed implicitly in interactional behaviors, such as turn‐taking patterns (Zeegers et al. 2017). Indeed, a recent review further supports this link, showing co‐activation of mirror and mentalizing systems (frontal and temporo‐parietal areas) in the brain during vocal turn‐taking interactions in adults (Kelsen et al. 2022). Recent work has begun to examine this association. For example, Foley et al. (2022) found that prenatal parental “mind‐mindedness” predicts the frequency of conversational turns at 7 months postpartum. Notably, whereas maternal prenatal mind‐mindedness was associated with overall levels of family talk, paternal prenatal mind‐mindedness was specifically related to more frequent infant‐initiated (but not parent‐initiated) exchanges. These findings suggest that mentalizing may play a foundational role in shaping early turn‐taking behavior. However, this study relied on global measures of conversational frequency and did not capture the temporal microstructure of turn‐taking, such as response latencies or overlaps. Moreover, the developmental trajectory of these fine‐grained processes, particularly in father‐infant dyads, remains underexplored. In addition, while infants tend to be less responsive to fathers than to mothers, potentially due to fathers' traditional role as secondary caregivers (Boiteau et al. 2021), it remains unknown whether father‐infant interaction time shapes the development or quality of turn‐taking.

### 1.1 The Present Study

The aim of the present study was to address the gaps in literature as outlined above by investigating the development of turn‐taking in infant‐father interactions during the first 6 months of life. Specifically, we set out to: (1) examine how vocal turn‐taking behaviors develop and change over the first 6 months of infancy; and (2) explore whether turn‐taking behaviors are influenced by paternal characteristics, such as mentalizing skills and involvement. We employed a longitudinal study design, allowing us to track changes in turn‐taking behavior over time and examine their potential predictors. In this study, vocal turn‐taking was operationalized by the number of turns taken by the father and infant in relation to their overall vocalizations, by the number and duration of overlapping vocalizations, and turn‐latencies. We investigated the following hypotheses:

H 1 Turn‐taking in infant–father interactions becomes more coordinated during the first 6 months of an infant's life, indicated by an increase in the number of turn‐taking sequences, shorter response latencies, fewer overlaps, and shorter overlap durations over time.

H 2 More complex infant turn‐taking at 6 months (i.e., more turns, shorter response latencies, fewer overlaps, and shorter overlap durations) is positively associated with higher paternal mentalizing skills and greater paternal involvement.

## 2 Methods

### 2.1 Participants

The current study included a sample of *N* = 39 healthy fathers (*M*_age_ = 33; *range* = 21–52) and their first‐born children (31% girls), who participated in a larger longitudinal study. Children had a mean age of 7.6 weeks (*range* = 5–12) at the first visit (T1) and a mean age of 6.4 months (*range* = 5–8) at the second visit (T2). Sample size was determined for the larger study. A priori power analysis using G*power yielded a sample size of *N* = 45 at 90% power. Participating families were recruited mainly through Facebook advertisements, birth information events at the local hospital, and flyers left with midwives and gynecologists. Participation was voluntary and financially compensated. Parents signed an informed consent for their own and their infant's participation prior to enrollment and could withdraw from the study at any time. Infants were born healthy and at term (gestation week: *M* = 39.6; birth weight: *M* = 3227g). Families ranked as middle to high SES, based on education and family income (see Table 1 for sample characteristics).

**TABLE 1:** Descriptive statistics of demographics for fathers and infants.

	Included (*N* = 39)	Drop‐out/excluded (*n* = 6)
	*M* (SD)	*M* (SD)
Father age (years)	32.9 (5.84)	33.7 (6.31)
Infant age T1 (weeks)	7.6 (1.50)	—
Infant age T2 (months)	6.4 (0.68)	—

	*N* (%)	*N* (%)
Nationality		
Country 1 Austria	34 (87.2%)	3 (50.0%)
Country 2 Germany	5 (12.8%)	2 (33.3%)
Other	—	1 (16.7%)
Education		
Apprenticeship	4 (10.3%)	—
A‐levels (i.e., high school)	6 (15.4%)	3 (50.0%)
University degree	27 (69.2%)	2 (33.3%)
Not specified	2 (5.1%)	1 (16.7%)
Family income per year		
Low (< 32.000€)	2 (5.1%)	—
Lower middle (32.000€–48.000€)	9 (23.1%)	2 (33.3%)
Middle (48.000€–67.000€)	16 (41.0%)	4 (66.7%)
Upper middle/high (> 67.000€)	12 (30.8%)	—

The sample initially consisted of *N* = 45 German speaking first‐time parents. The participation rate from baseline to the first assessment with the infant (T1) was 89% (*n* = 40). In addition, one father‐child dyad had to be excluded post hoc because the father spoke a different language with his infant at both times. Dyads included in the final sample did not differ from those excluded on any of the assessed sociodemographic characteristics (see Table 1 and Supporting Information S1: Figures S1–S3). General inclusion criteria for study participation were German language proficiency, cohabitation with spouse, first‐time parenthood, and carrying a single embryo to ensure compatibility and reduce confounding factors. Severe mental health problems were an exclusion criterion.

### 2.2 Procedure

**FIGURE 1:**
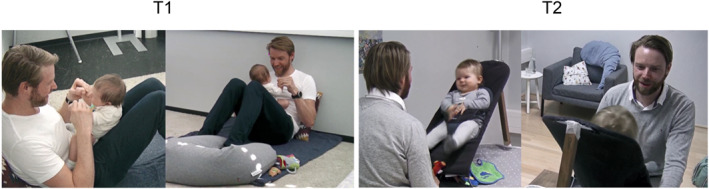
Illustration of father's and infant's position during recording in the laboratory at T1 and T2. Printed with permission.

Data for this study was drawn from a larger longitudinal investigation including five timepoints. For the purpose of the present study, we used two time points. The first assessment (T1) was 6–8 weeks after birth and involved a 20‐min video observation of a father‐child interaction recorded in a laboratory setting, when infants were relaxed and alert. The father and infant were positioned facing each other to provide a basis for communication. Fathers, seated on a blanket on the floor, were instructed to place the infants either on a pillow in front of them or on their bent thighs. The video recording was stopped prematurely or interrupted if the infant cried excessively or fell asleep. The first uninterrupted 3 minutes (i.e., infant was not crying or sleeping but in a quiet awake state) of each interaction were used for the analysis of paternal and infant vocalizations. This procedure was repeated 6 months after birth (T2), when fathers sat in a chair facing their infants placed in a fixed bouncer (see Figure 1 for an illustration of the interaction settings). If the infants did not like sitting in the bouncer (∼30%), they could switch to a face‐to‐face position on the floor. Additionally, the Parent Development Interview was administered, which took approximately 30–90 minutes to complete (PDI‐R; Slade et al. 2022). While fathers were interviewed, infants remained with their mothers. All procedures and measures of the larger longitudinal study were approved by the local ethics committee.

### 2.3 Measures

#### 2.3.1 Vocalizations

Two trained assistants coded father and infant vocalizations (excluding crying) based on audiotape and visualized sonograms using ELAN software (version 6.4). Following Gratier et al. (2015), vocalizations were defined as continuous vocal sound production that differed from vegetative sounds, such as coughing, burping, growling, or other environmental sounds. Only vocalizations but not vegetative sounds were coded in this study. Inter‐rater reliability was assessed on 20% of data, and agreement between the two raters was high (T1: infant vocalizations *κ* = 0.97, paternal vocalizations *κ* = 0.95; T2: infant vocalizations *κ* = 0.95, paternal vocalizations *κ* = 0.93).

We calculated turn‐taking sequences as paternal vocalizations followed by infant vocalizations, and vice versa, within 3000 ms (see e.g., Boiteau et al. 2021; Gratier et al. 2015; T. Nguyen et al. 2023). We assessed the total number of turn‐taking sequences, the number of turns taken by the infant or the father as well as their respective response time, and the number and duration of instances where the infant or father overlapped their vocalizations with the preceding vocalization of the other partner. For data analysis, we used the proportion of turns and overlaps relative to each partner's total number of vocalizations, as well as individual and total turn‐response latency and overlap duration (in seconds), as indicators of turn‐taking. Table 2 provides an overview of turn‐taking variables used in this study along with their detailed descriptions. To examine the robustness of findings across different operationalizations of vocal contingency, we additionally conducted sensitivity analyses using 1‐s and 2‐s time windows.

**TABLE 2:** Description of turn‐taking indices.

Label	Description
Infant turns	Proportion of turns initiated by infant
Father turns	Proportion of turns initiated by father
Total turns	Proportion of overall turns
Infant latency	Infant turn latency (response time in seconds)
Father latency	Father turn latency (response time in seconds)
Total latency	Total turn latency (response time in seconds)
Infant overlaps	Proportion of overlaps by infant
Father overlaps	Proportion of overlaps by father
Total overlaps	Proportion of overall overlaps
Infant overlap duration	Average duration (seconds) of infant overlaps
Father overlap duration	Average duration (seconds) of father overlaps

#### 2.3.2 Reflective Functioning Assessed With PDI

Paternal mentalizing skills, operationalized as Reflective Functioning (RF), were measured using the Reflective Functioning Scale (Fonagy et al. 1998) applied to the Parent Development Interview (PDI; Slade et al. 2022) conducted at T2. The applied short version of the PDI asks 15 questions about the parent's current and developing relationship with their child. The interview was audiotaped and transcribed verbatim for further analyses. RF was scored by a reliable rater on an 11‐point scale ranging from −1 (i.e., negative RF indicates anti‐reflective stances and responses that are hostile, bizarre or inappropriate) to 9 (i.e., full or exceptional RF, describing extremely elaborated and sophisticated reflection; Fonagy et al. 1998). Internal consistency of the RF scale is generally considered as high (Cronbach's alpha = 0.91; Sleed et al. 2020). Inter‐rater reliability between two raters was assessed on 20% of the data, with inter‐rater agreement of ICC = 0.62. In cases of disagreement, consensus ratings were used for further analysis. All raters were blind to the characteristics of the sample.

#### 2.3.3 Father's Time Spent With Infant

To obtain a measure of paternal active involvement and presence as interaction partners for their children in the first 6 months, we asked fathers how many weeks and when they stayed at home with their baby. This included time off work, daddy's month (1‐month unpaid additional leave for fathers after childbirth), paternity leave or individual solutions. In the current sample, fathers stayed at home approximately 4.6 weeks. Austrias national average of leave for fathers after birth is approximately 9 days (Reiter and Spitzer 2026).

#### 2.3.4 Data Analyses

Data analyses were conducted in R (v.4.2.2; R Core Team 2021). Approximately 18% of the data from *n* = 78 infant‐father interaction recordings (across T1 and T2) were excluded from analysis due to issues with codability. This was primarily caused by infants falling asleep, crying, or deviations from the structured face‐to‐face interaction (e.g., fathers moving around the room to soothe their child). To assess the nature of missing data, Little's MCAR test was conducted, using the “naniar” package (Tierney and Cook 2023), showing that the data were missing completely at random (*χ* ^*2*^ = 31.6, df = 34, *p* = 0.584). This allowed for the use of multiple imputation to handle missing values, which was carried out using the “mice” package (Buuren and Groothuis‐Oudshoorn 2011).

Given the non‐normal distribution of infant vocalizations at both timepoints and most of the turn‐taking variables (see Supporting Information S1: Tables S1 and S2), outliers were addressed using a percentile‐based approach. To account for variability in overall vocalization frequency across father‐infant dyads, all turn‐taking measures were calculated as proportions relative to the total number of vocalizations. These percentage‐based calculations were used in the subsequent analyses to ensure comparability across dyads.

To examine associations between turn‐taking variables across time points, Pearson correlations were computed using the Hmisc package (Harrell 2026), with *p*‐values adjusted for multiple testing using the Holm correction. To track changes in turn‐taking between infants and fathers over time, Wilcoxon signed‐rank tests were used to evaluate differences in various turn‐taking indices across time points (T1, T2). Given the non‐normal distribution of data and the small sample size, this non‐parametric approach was selected to ensure robust statistical inference.

To further explore the relationships between time, paternal characteristics (i.e., RF, time spent at home), and turn‐taking behaviors, mixed‐effects models were employed using the lme4 package (Bates et al. 2015). These models accounted for the nested structure of the data (repeated measures within dyads) and included random intercepts for dyads to capture variability between individuals. Each model incorporated time (T1, T2) as a fixed effect, as well as paternal characteristics (i.e., reflective functioning and involvement) as predictors, with interaction terms to examine potential moderation effects. Outcome variables included the turn‐taking measures (see Table 2) and their subcomponents.

Outcome ∼ time + RF + paternal involvement + time*RF + time*paternal involvement + (1 | ID).

Model fit was assessed using conditional and marginal *R* ^2^, which provided insights into the variance explained by fixed effects alone and by the combination of fixed and random effects, respectively. To examine robustness, additional models including paternal age and infant sex as covariates were estimated.

## 3 Results

### 3.1 Descriptive Analysis of Paternal and Infant Vocalizations

Descriptive statistics of the main study variables are reported in Table 3. There was high variability in overall vocalizations between dyads ranging from 2 to 47 for infant vocalizations and between 2 and 83 for paternal. Already at 7 weeks, infants initiated on average 7.6 turn‐taking sequences and 47% of their overall vocalizations were involved in turn‐taking. At this age, fathers initiated 7.7 turn‐taking sequences and 16% of their vocalizations were involved in turn‐taking. At 6 months, overall vocalizations were again variable between dyads. Infant vocalizations remained similar between T1 and T2 (*Z* = −1.12; *p* = 0.261), while fathers vocalized more at T2 compared to T1 (*Z* = −2.30; *p* = 0.022).

**TABLE 3:** Descriptive statistics of study variables.

	T1				T2				
Variable	*M* (SD)	*Md*	*Min*	*Max*	*M* (SD)	*Md*	*Min*	*Max*	*p*
IV	13.59 (9.5)	10.00	2	47	15.49 (9.3)	13.00	2	32	0.261
FV	40.67 (20.4)	39.00	2	83	49.38 (16.1)	53.00	12	80	0.022
IT	7.62 (6.6)	6.00	0	21	5.46 (3.8)	5.00	0	15	0.014
FT	7.67 (6.8)	5.00	0	25	7.10 (4.2)	6.00	0	17	0.681
IL	0.91 (0.50)	0.84	0.15	2.12	0.86 (0.3)	0.86	0.10	1.78	0.856
FL	0.92 (0.6)	0.79	0.29	2.56	0.80 (0.4)	0.74	0.06	1.67	0.295
IO	2.72 (3.6)	1.00	0	13	3.95 (4.7)	2.00	0	24	0.245
FO	1.18 (1.9)	0.00	0	7	2.62 (2.6)	2.00	0	10	0.005
IOD	0.41 (0.1)	0.44	0.15	0.68	0.49 (0.3)	0.36	0.01	1.60	0.315
FOD	0.41 (0.3)	0.28	0.13	0.95	0.52 (0.5)	0.36	0.01	1.57	0.365

*Note:* This table presents the absolute frequencies of vocalizations (IV = infant vocalizations; IF = father vocalizations), turn‐taking (IT = infant turns; FT father turns), latencies (IL = infant latency; FL = father latency) and overlap indices (IO = infant overlaps; FO = father overlaps; IOD = infant overlap duration; FOD = father overlap duration). Turn latencies and overlap durations are presented in seconds. For subsequent analyses, proportions relative to the total number of vocalizations were used.

Bivariate correlations revealed no significant associations between turn‐taking initiated by fathers nor infants at neither T1 nor T2. While there was a strong positive correlation between infant‐initiated turns at T1 and T2 (*r* = 0.548, *p* < 0.001), we found no significant relationships in turn‐taking behavior of fathers between these two timepoints. Furthermore, no significant associations were found between infant and father response latencies within timepoints. A full correlation matrix is provided in the supplements (see Supporting Information S1: Table S3).

### 3.2 Development of Vocal Turn‐Taking Behavior Between Infants and Their Fathers in the First 6 months of Life

Results indicated a significant decrease in the proportion of turns initiated by infants between T1 and T2 (*V* = 515.5; *p* = 0.014). The proportion of overlaps initiated by fathers showed a significant increase (*Mdn*_*t2*_ = 1.45; *Mdn*_*t3*_ = 4; *V* = 123; *p* = 0.005) between T1 and T2. No other indices showed significant changes between time points (all *p* > 0005; see Table 4).

**TABLE 4:** Results of wilcoxon signed‐rank tests on turn‐taking variables.

Variable	V statistic	*p*
Infant turns	515.5	0.014
Father turns	419	0.694
Total turns	432	0.566
Infant latency	403	0.863
Father latency	451	0.403
Total latency	442	0.477
Infant overlaps	259	0.248
Father overlaps	123	0.005
Total overlaps	238	0.056
Infant overlap duration	318	0.318
Father overlap duration	308	0.369

To examine whether findings depended on the operationalization of vocal contingency, we repeated the analyses using stricter time windows between turns of 1 and 2 s. Overall, the pattern of results was broadly comparable across time windows, especially for father‐initiated turns, while decreases in infant‐initiated turns and changes in total turns were more pronounced under narrower temporal thresholds (see Supporting Information S1: Table S4).

### 3.3 Associations of Turn‐Taking Behavior With Paternal Characteristics

#### 3.3.1 Proportions of Turn‐Taking

We found no significant effects of mentalizing or paternal involvement on proportions of overall turn‐taking (Total Turns), with fixed effects explaining only a small fraction of variability (marginal *R* ^*2*^ = 0.018). The model revealed that 48% of the variability in turn‐taking proportion was due to between‐dyad differences, as reflected by the large random intercept variance (*τ*_*00*_ = 64.22). Additionally, the residual variance (*σ* ^*2*^ = 68.46) indicates substantial within‐dyad variability over time, not accounted for by the fixed effects. Detailed results are presented in Table 5. Similar results were found when analyzing infant and father turns separately (see Supporting Information S1: Tables S5 and S6 for further detailed results).

**TABLE 5:** Mixed‐effects model for total turns.

*Predictors*	Total turns				
	*Estimate*	*S.E.*	CI	*t*	*p*
(Intercept)	20.95	7.03	6.92–34.97	2.98	0.004
Time	5.58	7.14	−8.67 to 19.82	0.78	0.438
RF	−0.52	1.19	−2.88 to 1.85	−0.44	0.664
Paternal involvement	0.00	0.61	−1.21 to 1.21	0.00	0.998
Time × RF	−0.45	1.20	−2.85 to 1.95	−0.37	0.710
Time × paternal involvement	−0.48	0.62	−1.71 to 0.75	−0.78	0.440
Random effects					
σ^2^	68.46				
τ_00 ID_	64.22				
ICC	0.48				
N _ID_	39				
Observations	78				
Marginal *R* ^2^/Conditional *R* ^2^	0.018/0.493				

*Note:* Total turns ∼ time + RF + paternal involvement + time*RF + time*paternal involvement + (1 | ID).

#### 3.3.2 Turn Latency

Mixed effect models showed no significant effects of time, RF and paternal involvement on the latency of turn‐taking behavior (*p*s > 0.05). The small residual variance (*σ* ^*2*^ = 0.10) and near‐zero random intercept variance (*τ*_*00*_ ∼ 0.00) indicate minimal individual differences and relative stability in turn latency across dyads and over time (see Supporting Information S1: Tables S7–S9).

#### 3.3.3 Overlap

Paternal RF was significantly associated with fewer father overlaps, *b* = −1.12; SE = 0.44, 95% CI [−2.00; −0.25]; *t* = −2.56; *p* = 0.013, while paternal involvement showed no significant effect, *b* = −0.24; SE = 0.22; 95% CI [−0.68; 0.21]; *t* = −1.05; *p* = 0.298. Random effects showed a residual variance (*σ* ^*2*^ = 15.05) and a random intercept variance for dyads (*τ*_*00*_ = 3.08), with 17% of variance in overlaps attributed to between‐dyad differences. No significant effects were found for infant overlaps. See detailed results of these models in Supporting Information S1: Tables S10–S12.

#### 3.3.4 Overlap Duration

Paternal RF was significantly associated with infant overlap duration, *b* = 0.05; SE = 0.02; 95%CI [0.00; 0.09]; *t* = 2.00; *p* = 0.050, indicating an increase in overlap duration for infants when fathers scored higher on mentalizing skills (see Supporting Information S1: Table S13 for detailed results). Additionally, there was a significant interaction effect between time and RF, *b* = −0.07; SE = 0.03; 95% CI [−0.14; −0.00]; *t* = −2.10; *p* = 0.039, suggesting that the association between mentalizing skills and overlap duration weakens over time. Paternal involvement did not significantly relate to overlap duration, *b* = 0.01; SE = 0.01; *95%*CI [−0.01; 0.04]; *t* = 1.15; *p* = 0.255. Random effects showed residual variance (*σ* ^*2*^ = 0.05), with no additional variance attributable to dyads (*τ*_*00*_ = 0.00). Fixed effects explained 8.1% of the variance in infant overlap duration (marginal *R* ^*2*^ = 0.081).

In contrast, paternal involvement was significantly associated with shorter paternal overlap duration, *b* = −0.05; SE = 0.02, 95% CI [−0.09; −0.01]; *t* = −2.55; *p* = 0.013 (see Supporting Information S1: Table S14). No other predictors showed significant effects. Random effects indicated a residual variance (*σ* ^*2*^ = 0.14) with no variance attributable to dyads (*τ*_*00*_ = 0.00). Fixed effects accounted for 12.1% of the variance in father overlap duration (marginal *R* ^*2*^ = 0.121).

Including paternal age and infant sex as covariates did not substantially alter the patterns of results across turn‐taking variables (see Supporting Information S1: Tables S15–S25 for detailed results). In addition, sensitivity analyses using alternative time windows of 1 and 2s between turns demonstrated a largely comparable pattern of findings to the primary analyses based on the 3s window (see Supporting Information S1: Tables S26 and S27 for detailed results).

## 4 Discussion

In this study, we explored vocal turn‐taking between fathers and their infants within the first 6 months of life, investigating its development and associations with paternal characteristics as well as caregiving behaviors. Our findings indicate that already by 7 weeks of age, distinct turn‐taking patterns exist in father‐infant interactions. Overall, we observed little change in turn‐taking indices over time, but strong evidence of large differences between individual dyads. Given the necessity of precise timing in turn‐taking, particularly the ability to anticipate the end of a partner's vocalization, we explored whether paternal mentalizing skills (i.e., the ability to infer others' mental and emotional states) contributed to these turn‐taking patterns. While higher paternal mentalizing ability was associated with less paternal vocal overlap, other aspects, such as shorter switching pauses and decreased overlap duration, showed no significant relations with mentalizing capacity. Moreover, paternal overall involvement in the infant's life was associated with shorter overlap durations. Taken together, our results suggest that father‐infant turn‐taking largely parallels patterns observed in mother‐infant dyads, albeit sometimes at lower frequencies, suggesting that early communicative exchanges should be understood within a more neutral conceptualization of parenthood (see Fagan et al. 2014).

Our data showed that nearly half of infant vocalizations at 7 weeks were involved in turn‐taking sequences. This early presence of coordinated exchanges may reflect infants' strong reliance on social partners, particularly faces, for interaction at this developmental stage (e.g., Frank et al. 2014). Furthermore, we found that fathers and infants equally often initiated turn‐taking with no significant change over time, mirroring results from mother‐infant dyads (Gratier et al. 2015). Overall, we found no changes in turn‐taking behavior over time, with two exceptions. Contrary to early assumptions that turn‐taking competence increases with age (e.g., Ginsburg and Kilbourne 1988), we found an overall decrease in infant turn‐taking. This is consistent with more recent findings showing no change or a decrease in infant turn‐taking behaviors in interactions with mothers between 2 and 5 months of age (Gratier et al. 2015; Hilbrink et al. 2015). One possible explanation could be that while general turn‐taking abilities may indeed improve with age, older infants begin to engage in more multimodal forms of communication. By 6 months, infants are typically beginning to show indications of focused attention and begin shifting their focus more to objects (e.g., Cleveland et al. 2007), potentially leading to more frequent use of gaze, gestures, or object‐directed actions in turn‐taking, rather than relying solely on vocal exchanges. As a result, vocal turn‐taking might decrease even as broader communicative competence continues to develop. Interestingly, sensitivity analyses using alternative contingency windows (1 and 2s) suggested that some developmental effects in infant turn‐taking were stronger for stricter time windows between turns, indicating that developmental changes may be particularly evident in tightly coordinated exchanges. Moreover, the proportion of turn‐taking relative to total vocalizations was higher in Gratier et al. ’s (2015) study with mothers (79% and 62%) than in our sample with fathers (47% and 37%). It is possible that mothers, who typically spend more time interacting with their infants in the first half of their first year of life due to factors like maternity leave, breastfeeding and their socialization as primary caregiver (e.g., Kotila et al. 2013; Trinder 2025), are more likely to develop nuanced communication patterns, resulting in slightly higher turn‐taking rates. Overall, these findings suggest that while the development of turn‐taking between infants and their mothers and fathers may follow similar trajectories, turn‐taking with fathers might occur less frequently.

We also found more paternal overlap at 6 months of age than when infants were 7 weeks, violating the assumption that overlap decreases with infant maturation (e.g., Elias and Broerse 1996; Ginsburg and Kilbourne 1988; Hilbrink et al. 2015), but consistent with research investigating mothers (Gratier et al. 2015). The observed increase in paternal overlap with infant age may be a byproduct of increased overall paternal vocal activity, while infant vocalization rates remained relatively stable. As fathers vocalize more—potentially because they increasingly perceive their infants as communicative partners—the probability of unintentionally overlapping with infant vocalizations may rise simply due to greater paternal vocal output. Supporting this interpretation, recent home‐based observations showed that as infants grow, parents respond more frequently to their vocalizations (Lee and Ha 2023), suggesting a developmental shift in how caregivers perceive and engage with infants.

Our results also showed that response latency remains stable across the first 6 months for both infants and fathers. While this is consistent with most of the literature indicating stability in caregiver latency (e.g., Gratier et al. 2015; Hilbrink et al. 2015), for infants evidence is mixed. Some studies report an increase in infant response latency, although the reported onset of this change varies considerably, from as early as 4–5 months (e.g., Gratier et al. 2015) to around 9 months (see Hilbrink et al. 2015; Levinson 2016). A recent meta‐analysis further supports this general developmental trend, while also identifying a peak in response latency around 20 months of age (V. Nguyen et al. 2022). Although these findings consistently indicate that infant latency increases with age, they differ on when this change first emerges. Our study did not find evidence of an increase within the first 6 months of life, but the limited number of assessed timepoints precludes strong conclusions about developmental changes beyond this period. These findings underscore the importance of considering both caregiver and infant behaviors over time, highlighting nuanced developmental trajectories in turn‐taking and the need for further research to clarify patterns of overlap and response latency across early infancy.

Surprisingly we found a strong association between infants' turn‐taking at 7 weeks and 6 months, suggesting stability in early communicative patterns. In contrast, no such association was found for fathers. This seems to suggest that different mechanisms underlie turn‐taking behavior in infants and fathers. It is possible that once infants acquire a foundational communicative skill such as vocal turn‐taking, they continue to build on and use it consistently over time. Fathers, however, may exhibit more situational and variable engagement in vocal turn‐taking. Their interaction style might shift over time, for example, with increasing infant age, fathers may engage more through physical play or object‐focused interaction (e.g., Amodia‐Bidakowska et al. 2020; Crawley and Sherrod 1984) rather than vocal exchanges alone. This could reduce the consistency of vocal turn‐taking across time, potentially explaining the lack of correlation. Consequently, vocal turn‐taking may not fully capture the evolving nature of paternal interaction, highlighting the need for a more multimodal assessment approach that includes nonverbal behaviors such as gestures, gaze, and shared object play (e.g., Kendrick et al. 2023; Rohlfing et al. 2020). Alternatively, contextual factors such as fathers' mood, or situational distractions during recording could also contribute to the observed inconsistency in paternal turn‐taking behavior. Indeed, previous research has shown that maternal depressive symptoms were associated with slower responses to infants' vocalizations (e.g., Smith et al. 2023), suggesting that caregivers' mood or emotional state may have an effect on the temporal dynamics of turn‐taking.

We found large variability in the amount of turn‐taking, and results from the mixed effect models suggest large inter‐individual differences in turn‐taking behavior. While this is consistent with recent findings showing differences in turn‐taking perception/skills as early as 6 months of age based on different family characteristics (e.g., socioeconomic status, presence of siblings; Meunier et al. 2024), in the present study we only included first‐time parents and the sample was relatively homogeneous in terms of socioeconomic status. Therefore, there may be some additional factors explaining inter‐individual differences in turn taking that we did not account for in our study, such as mood for conversation, time of day, how familiar fathers and their infants were with each other, or how involved fathers were in caregiving. In fact, we approximated paternal involvement during the first 6 months of the infant's life by assessing the amount of time they spent on leave from work during this period; however, this variable was a significant predictor only for paternal overlap duration, but not for any of the other turn‐taking indices. It is possible that turn‐taking is not dependent on caregiver involvement but rather reflects a more universal human capacity (Levinson 2016; Stivers et al. 2009) that is also present in interactions with strangers (e.g., Crown et al. 2002). Alternatively, paternal involvement may be indicative of better turn‐taking structures, but our operationalization of this variable in the present study was too vague. Indeed, there is an ongoing debate in fatherhood research regarding accurate operationalizations of paternal involvement and whether more qualitative and multidimensional aspects are relevant than simply the amount of time fathers spend with their children (e.g., Cabrera 2020; Diniz et al. 2023; Palkovitz 2019). Thus, future research needs to incorporate additional qualitative indices of paternal involvement and include interactions with a stranger to better understand the unique characteristics of infant‐father turn‐taking behavior.

Given the limited understanding of the mechanisms underlying turn‐taking (Cosper and Pika 2025), we further explored paternal mentalizing skills as a potential contributor to turn‐taking behavior, based on the assumption that the ability to think about and understand others' feelings, intentions and behavior could be a pivotal ability for being a skilled turn‐taker. Our findings partially supported this assumption. Specifically, at 6 months, higher paternal RF was associated with a lower proportion of father‐infant overlap, suggesting that mentalizing abilities may enhance fathers' capacity to accurately predict the end of infant vocalizations and thereby reduce overlapping turns. At the same time, higher paternal RF was associated with longer infant overlap duration. Although these findings may initially appear contradictory, they may reflect distinct processes captured by overlap frequency versus overlap duration in early proto‐conversations, overlaps are not necessarily disruptive but may occur during periods of heightened infant arousal and communicative engagement (e.g., Wass et al. 2022). Thus, fewer paternal overlaps may indicate greater paternal sensitivity to conversational timing, whereas longer infant overlaps may reflect infants' increasing participation within a mutually coordinated interactional exchange. Nevertheless, paternal RF was not associated with other turn‐taking indices, suggesting that its role in early vocal coordination may be selective or modest. Future research is needed to further clarify these associations and examine additional modalities of interaction. For example, fathers have been shown to use fewer vocalizations but more gestures than mothers (Morelli et al. 2022), raising the possibility that paternal mentalizing capacities may also manifest in non‐vocal forms of communicative coordination that were not captured in the present study.

### 4.1 Limitations

To the best of our knowledge, this is the first study to examine the development of turn‐taking in early father‐infant interactions, but several limitations must be acknowledged. First, the study setting introduced constraints to the father‐infant interaction, as we were unable to control for time of day or mood during filming. Due to fathers' work schedules, filming often took place in the evening when infants may not have been at their most content or alert state. Additionally, since fathers often interacted with their infants following prior sessions with mothers, infants were sometimes tired or less receptive to further engagement. As a result, approximately 18% of recordings could not be used for coding due to infants' uncooperative states (e.g., crying, restlessness, falling asleep), necessitating data imputation. While this approach yields more accurate and less biased estimates than simply excluding incomplete cases (Enders 2017) and imputations were conducted only after checking the randomness of missing data, the use of multiple imputation nonetheless introduces a potential source of bias. Fluctuations in mood were evident even within the analyzed recordings, with fathers or infants occasionally appearing tired or disengaged. Furthermore, while we instructed fathers to interact with their infants, we did not specifically emphasize vocal communication. For example, the presence of toys in the lab, although intended to contribute to a more natural interaction environment, could have led fathers to shift their focus to object‐focused play rather than verbal exchanges, which may have altered the typical patterns of communication and impacted the consistency of turn‐taking behaviors across recordings. In fact, Sosa (2016) found that parents of 10‐ to 16‐month‐old infants used fewer words during play with traditional toys compared to book‐sharing. However, the authors also reported no significant difference in the number of conversational turns across conditions. Conversely, toy engagement may also stimulate interaction and vocalizations; for instance, Miller et al. (2017) demonstrated that traditional toys could enhance infant vocalizations and elicit more parental responses. It may be that toys promote multimodal forms of turn‐taking beyond vocal exchanges, such as gaze shifts, gestures (e.g., pointing) or object manipulation, all of which we did not assess in the present study. Future research should therefore examine multimodal turn‐taking during toy‐mediated interactions to draw more comprehensive conclusions about early communicative dynamics.

Second, although key study variables were measured using best‐practice methods (e.g., second‐by‐second vocal coding, interviews for paternal mentalizing), paternal involvement was operationalized as the number of weeks fathers stayed at home after birth. While this measure provided a general estimate of fathers' time with their infants, it did not capture the quality, intensity, or context of father‐infant interactions, including engagement during evenings or weekends, which might be high in engaged fathers not formally on leave. Given that paternal involvement is a multifaceted construct that quantitative home hours cannot fully capture (see Fagan et al. 2014 for a discussion of the father involvement construct), future research would benefit from more fine‐grained assessments, such as interaction diaries or observational assessments of engagement quality (Cabrera 2020; Voyer‐Perron et al. 2024), to better understand how paternal involvement relates to the development of turn‐taking development.

Third, paternal reflective functioning was assessed only at T2. Although RF is generally considered relatively stable across shorter periods of time (Katznelson 2014), repeated assessments could provide important insights into potential changes associated with infant age and paternal involvement (e.g., Foley et al. 2022).

Finally, the study's small, relatively homogenous sample of fathers with western backgrounds and medium to high socioeconomic status restricts the generalizability of our findings. Although our sample size aligns with or exceeds similar studies (e.g., Boiteau et al. 2021; Dominguez et al. 2016; Gratier et al. 2015), it remains limited for detecting medium or small effects. This limitation is further underscored by post hoc power analyses, which revealed low statistical power for the fixed effects estimated in our mixed‐effect models. Including a broader range of paternal roles in future research—such as stay‐at‐home fathers, non‐residential fathers, and fathers from diverse cultural and socioeconomic backgrounds—would enhance our understanding of variability in infant‐father turn‐taking trajectories. This is particularly relevant given cultural variations in temporal parameters (Stivers et al. 2009); accounting for such diversity would strengthen the generalizability of future findings.

### 4.2 Conclusion and Implications for Practice and Further Research

This study provides important contributions to the understanding of turn‐taking development in early parent‐infant interactions. A recent review by Cosper and Pika (2025) highlighted the paucity of longitudinal research on turn‐taking. Our study addresses this gap by presenting longitudinal data on father‐infant interactions, providing novel insights into the developmental trajectory of turn‐taking in this understudied population. Our findings demonstrate that turn‐taking is not exclusive to mother‐infant interactions but emerges very early in father‐infant exchanges, highlighting the universality of this communicative behavior across caregiving contexts. Furthermore, this is the first study to investigate the role of paternal mentalizing in turn‐taking development. While exploratory in nature, the observed association between fathers' mentalizing abilities and reduced vocal overlap suggests that fostering mentalizing skills in young fathers may enhance not only their caregiving quality (e.g., Sourander et al. 2021) but also their communicative patterns with infants. Given the foundational role of early turn‐taking in language development (e.g., Donnelly and Kidd 2021; V. Nguyen et al. 2022), these findings underscore the potential benefits of supporting mentalizing as a target for intervention. Overall, these insights lay the groundwork for future research and interventions aimed at promoting early communicative behaviors and optimizing developmental outcomes through a deeper understanding of father‐infant interactions. Future research should continue to explore the nuanced dynamics of father‐infant interaction, including multimodal communication, to fully capture the complexity of early social development.

## Author Contributions

**Selina Ismair:** conceptualization, methodology, data curation, investigation, formal analysis, funding acquisition, visualization, project administration, writing – original draft, writing – review and editing. **Antonia Dinzinger:** conceptualization, writing – review and editing, funding acquisition, project administration, investigation. **Beate Priewasser:** resources, writing – review and editing. **Karl Heinz Brisch:** funding acquisition, writing – review and editing. **Gabriela Markova:** conceptualization, methodology, software, formal analysis, supervision, writing – review and editing, data curation.

## Ethics Statement

This study was conducted in accordance with the ethical standards outlined in the Declaration of Helsinki and received approval from the Ethics Committee of the Land Salzburg (Reference number: 415‐E/2217/8–2017).

## Conflicts of Interest

The authors declare no conflicts of interest.

## Supporting information


Supporting Information S1

## Data Availability

The data that support the findings of this study are available from the corresponding author upon reasonable request.
