# Supplementary material for: It's Daddy's Turn! Development of Turn‐Taking in Infant‐Father Interactions and Associations With Paternal Mentalizing and Involvement
Source: Infancy. 2026 Aug 10;31(4):e70115. doi: 10.1111/infa.70115 (PMC13457993; doi:10.1111/infa.70115)
Supplement: Supplementary file 1 [file INFA-31-0-s001.docx]

**Supplementary Material**

**Figure S1**

*Boxplot of Fathers’ Age by Group*

**Figure S2**

*Bar Chart of Fathers’ Annual Income by Group*

**Figure S3**

*Bar Chart of Fathers’ Education Level by Group*

**Table S1**

*Tests of Normality for Vocalization Variables*

| Variable | KS Statistic | KS *p* | SW Statistic | SW *p* |
| --- | --- | --- | --- | --- |
| Infant Vocalizations T1 | .186 | .002 | .882 | < .001 |
| Father Vocalizations T1 | .098 | .200 | .973 | .471 |
| Infant Vocalizations T2 | .144 | .041 | .925 | .013 |
| Father Vocalizations T2 | .124 | .136 | .980 | .689 |

Note. KS = Kolmogorov–Smirnov test; SW = Shapiro–Wilk test. p values below .05 indicate significant deviation from normality.

**Table S2**

*Test of Normality for Turn-Taking Variables*

| Variable | KS Statistic | KS *p* | SW Statistic | SW *p* |
| --- | --- | --- | --- | --- |
| Infant Turns T1 | .153 | .022 | .940 | .038 |
| Father Turns T1 | .115 | .200 | .927 | .014 |
| Total Turns T1 | .150 | .028 | .936 | .029 |
| Infant Turns T2 | .083 | .200 | .965 | .255 |
| Father Turns T2 | .092 | .200 | .958 | .156 |
| Total Turns T2 | .081 | .200 | .979 | .685 |
| Infant Latency T1 | .226 | < .001 | .871 | < .001 |
| Father Latency T1 | .142 | .047 | .824 | < .001 |
| Infant Overlaps T1 | .155 | .019 | .873 | < .001 |
| Father Overlaps T1 | .263 | < .001 | .749 | < .001 |
| Total Overlaps T1 | .224 | .001 | .890 | < .001 |
| Infant Overlaps T2 | .135 | .072 | .880 | .003 |
| Father Overlaps T2 | .150 | .028 | .877 | < .001 |
| Total Overlaps T2 | .215 | .002 | .892 | .003 |
| Infant Overlap Duration T1 | .106 | .200 | .965 | .257 |
| Father Overlap Duration T1 | .217 | < .001 | .824 | < .001 |

Note. KS = Kolmogorov–Smirnov test; SW = Shapiro–Wilk test. p values below .05 indicate significant deviation from normality.

**Table S3**

*Pearson Correlation Coefficients Matrix for Turn-Taking Variables at T1 and T2*

| Variable | 1. | 2. | 3. | 4. | 5. | 6. | 7. | 8. | 9. | 10. | 11. | 12. | 13. | 14. | 15. | 16. | 17. | 18. | 19. |
| --- | --- | --- | --- | --- | --- | --- | --- | --- | --- | --- | --- | --- | --- | --- | --- | --- | --- | --- | --- |
| 1. T1 TT |  |  |  |  |  |  |  |  |  |  |  |  |  |  |  |  |  |  |  |
| 2. T1 FT | .63** |  |  |  |  |  |  |  |  |  |  |  |  |  |  |  |  |  |  |
| 3. T1 IT | .20 | -.19 |  |  |  |  |  |  |  |  |  |  |  |  |  |  |  |  |  |
| 4. T2 TT | .53** | .25 | .33* |  |  |  |  |  |  |  |  |  |  |  |  |  |  |  |  |
| 5. T2 FT | .44** | .15 | .18 | .85** |  |  |  |  |  |  |  |  |  |  |  |  |  |  |  |
| 6. T2 IT | .30 | .08 | .55** | .53** | .12 |  |  |  |  |  |  |  |  |  |  |  |  |  |  |
| 7. T1 IL | -.03 | .27 | -.35* | -.05 | .04 | -.24 |  |  |  |  |  |  |  |  |  |  |  |  |  |
| 8. T1 FL | -.20 | -.18 | -.15 | -.28 | -.17 | -.28 | .18 |  |  |  |  |  |  |  |  |  |  |  |  |
| 9. T2 IL | .06 | .08 | .10 | .03 | .04 | .01 | .05 | -.28 |  |  |  |  |  |  |  |  |  |  |  |
| 10.T2 FL | -.04 | .10 | -.09 | -.04 | -.03 | -.22 | -.01 | -.08 | -.30 |  |  |  |  |  |  |  |  |  |  |
| 11.T1 TO | .38* | .17 | -.05 | .02 | -.07 | .05 | -.20 | -.27 | .06 | .04 |  |  |  |  |  |  |  |  |  |
| 12.T1 FO | .15 | .09 | .08 | .08 | .07 | -.03 | -.20 | -.28 | -.05 | .19 | .45** |  |  |  |  |  |  |  |  |
| 13.T1 IO | -.31 | -.17 | -.04 | -.29 | -.31 | -.11 | .06 | -.17 | -.13 | -.08 | .33* | .17 |  |  |  |  |  |  |  |
| 14.T2 TO | .32* | .21 | -.07 | .30 | .49** | -.25 | .10 | .10 | .02 | -.14 | -.00 | .08 | -.12 |  |  |  |  |  |  |
| 15.T2 FO | .30 | .17 | -.14 | .34* | .60** | -.26 | .11 | -.01 | .12 | -.25 | -.00 | .24 | -.15 | .80** |  |  |  |  |  |
| 16.T2 IO | .05 | .25 | -.08 | -.04 | .07 | -.31 | .27 | .11 | -.07 | .10 | -.14 | -.23 | -.04 | .64** | .22 |  |  |  |  |
| 17.T1 FOD | .14 | .10 | -.38* | .02 | .12 | -.09 | .42** | -.11 | -.09 | -.13 | .34* | .03 | .25 | .27 | .23 | .12 |  |  |  |
| 18.T1 IOD | .14 | -.20 | -.06 | .11 | .20 | -.14 | -.09 | .37* | .04 | -.16 | .03 | -.01 | -.39* | .14 | .17 | -.12 | .09 |  |  |
| 19.T2 FOD | -.11 | .00 | .17 | -.25 | -.21 | -.24 | .02 | .06 | -.05 | .47** | .19 | .22 | .24 | -.14 | -.22 | .05 | -.04 | .01 |  |
| 20.T2 IOD | -.25 | -.26 | -.03 | -.24 | -.32* | -.05 | -.15 | .06 | -.27 | .24 | .10 | .21 | .16 | -.05 | -.17 | .03 | -.19 | -.18 | .07 |

*Note.* **p* < .05. ***p* < .01. *p*-values were adjusted using the Holm correction. TT = total turn-taking; FT father turns; IT = infant turns; IL = infant latency; FL = father latency; TO = total overlaps; FO = father overlaps; IO = infant overlaps; FOD = father overlap duration; IOD = infant overlap duration.

**Table S4**
*Comparison of Wilcoxon Signed-Rank Test Results Across 1-, 2-, and 3-Second Contingency Windows*

|  | 3 s | | *1 s* | | *2 s* | |
| --- | --- | --- | --- | --- | --- | --- |
| Variable | V Statistic | *p* | *V Statistic* | *p* | *V Statistic* | *p* |
| Infant Turns | 515.5 | .014 | 549 | .021 | 498 | .123 |
| Father Turns | 419 | .694 | 451 | .399 | 495 | .144 |
| Total Turns | 432 | .566 | 562 | .015 | 545 | .029 |

*Note.* Wilcoxon signed-rank tests comparing turn-taking variables between T1 (6 weeks) and T2 (6 months) across different contingency-window definitions (1 s, 2 s, and 3 s). V = Wilcoxon signed-rank statistic.

**Table S5**

*Mixed-Effects Model For Infant Turns*

|  | **Proportion of Turn-Taking (TT_FI_prop)** | | | | |
| --- | --- | --- | --- | --- | --- |
| *Predictors* | *Estimates* | *se* | *CI* | *Statistic* | *p* |
| (Intercept) | 16.86 | 14.79 | -12.62 – 46.35 | 1.14 | 0.258 |
| Time (T1) | 13.66 | 14.46 | -15.18 – 42.51 | 0.94 | 0.348 |
| RF | 2.97 | 2.49 | -2.01 – 7.94 | 1.19 | 0.238 |
| Paternal Involvement | 1.58 | 1.28 | -0.97 – 4.13 | 1.24 | 0.220 |
| Time × RF | -0.54 | 2.44 | -5.41 – 4.32 | -0.22 | 0.825 |
| Time × Paternal Involvement | -0.32 | 1.25 | -2.81 – 2.17 | -0.26 | 0.798 |
| **Random Effects** | | | | | |
| σ^2^ | 280.64 | | | | |
| τ_00_ _ID_ | 305.85 | | | | |
| ICC | 0.52 | | | | |
| N _ID_ | 39 | | | | |
| Observations | 78 | | | | |
| Marginal R^2^ / Conditional R^2^ | 0.080 / 0.560 | | | | |

*Note.* *Father Turns ~ Time + RF + Paternal Involvement + Time*RF + Time*Paternal Involvement + (1 | ID)*.

**Table S6**

*Mixed-Effects Model For Father Turns*

|  | Father Turns | | | | |
| --- | --- | --- | --- | --- | --- |
| *Predictors* | *Estimates* | *se* | *CI* | *Statistic* | *p* |
| (Intercept) | 20.78 | 6.02 | 8.78 – 32.79 | 3.45 | 0.001 |
| Time (T1) | -3.01 | 7.80 | -18.58 – 12.55 | -0.39 | 0.700 |
| RF | -1.24 | 1.02 | -3.27 – 0.78 | -1.22 | 0.225 |
| Paternal Involvement | -0.15 | 0.52 | -1.18 – 0.89 | -0.28 | 0.779 |
| Time × RF | 1.52 | 1.32 | -1.10 – 4.15 | 1.16 | 0.251 |
| Time × Paternal Involvement | -0.45 | 0.67 | -1.80 – 0.89 | -0.67 | 0.505 |
| Random Effects | | | | | |
| σ^2^ | 81.66 | | | | |
| τ_00_ _ID_ | 15.53 | | | | |
| ICC | 0.16 | | | | |
| N _ID_ | 39 | | | | |
| Observations | 78 | | | | |
| Marginal R^2^ / Conditional R^2^ | 0.048 / 0.200 | | | | |

*Note.* *Infant Turns ~ Time + RF + Paternal Involvement + Time*RF + Time*Paternal Involvement + (1 | ID)*.

**Table S7**

*Mixed-Effects Model For Total Turn-Taking Latency*

|  | Latency | | | | |
| --- | --- | --- | --- | --- | --- |
| *Predictors* | *Estimates* | *se* | *CI* | *Statistic* | *p* |
| (Intercept) | 0.93 | 0.20 | 0.53 – 1.32 | 4.69 | <0.001 |
| Time (T1) | 0.32 | 0.28 | -0.24 – 0.87 | 1.13 | 0.264 |
| RF | -0.00 | 0.03 | -0.07 – 0.06 | -0.09 | 0.932 |
| Paternal Involvement | -0.02 | 0.02 | -0.05 – 0.02 | -1.01 | 0.315 |
| Time × RF | -0.07 | 0.05 | -0.16 – 0.02 | -1.49 | 0.142 |
| Time × Paternal Involvement | 0.01 | 0.02 | -0.04 – 0.06 | 0.47 | 0.641 |
| Random Effects | | | | | |
| σ^2^ | 0.10 | | | | |
| τ_00_ _ID_ | 0.00 | | | | |
| N _ID_ | 39 | | | | |
| Observations | 78 | | | | |
| Marginal R^2^ / Conditional R^2^ | 0.082 / NA | | | | |

*Note.* *Latency ~ Time + RF + Paternal Involvement + Time*RF + Time*Paternal Involvement + (1 | ID)*.

**Table S8**

*Mixed-Effects Model For Infant Turn-Taking Latency*

|  | Infant Latency | | | | |
| --- | --- | --- | --- | --- | --- |
| *Predictors* | *Estimates* | *se* | *CI* | *Statistic* | *p* |
| (Intercept) | 0.99 | 0.25 | 0.49 – 1.48 | 3.99 | <0.001 |
| Time (T1) | 0.26 | 0.34 | -0.43 – 0.95 | 0.75 | 0.453 |
| RF | -0.02 | 0.04 | -0.10 – 0.07 | -0.40 | 0.690 |
| Paternal Involvement | -0.01 | 0.02 | -0.06 – 0.03 | -0.66 | 0.513 |
| Time × RF | -0.05 | 0.06 | -0.17 – 0.06 | -0.92 | 0.363 |
| Time × Paternal Involvement | 0.01 | 0.03 | -0.05 – 0.07 | 0.21 | 0.836 |
| Random Effects | | | | | |
| σ^2^ | 0.16 | | | | |
| τ_00_ _ID_ | 0.01 | | | | |
| ICC | 0.03 | | | | |
| N _ID_ | 39 | | | | |
| Observations | 78 | | | | |
| Marginal R^2^ / Conditional R^2^ | 0.046 / 0.076 | | | | |

*Note.* *Infant Latency ~ Time + RF + Paternal Involvement + Time*RF + Time*Paternal Involvement + (1 | ID)*.

**Table S9**

*Mixed-Effects Model For Father Turn-Taking Latency*

|  | Father Latency | | | | |
| --- | --- | --- | --- | --- | --- |
| *Predictors* | *Estimates* | *se* | *CI* | *Statistic* | *p* |
| (Intercept) | 0.84 | 0.32 | 0.21 – 1.47 | 2.65 | 0.010 |
| Time (T1) | 0.19 | 0.45 | -0.70 – 1.09 | 0.43 | 0.666 |
| RF | 0.03 | 0.05 | -0.08 – 0.13 | 0.48 | 0.630 |
| Paternal Involvement | -0.03 | 0.03 | -0.09 – 0.02 | -1.21 | 0.231 |
| Time × RF | -0.04 | 0.08 | -0.19 – 0.11 | -0.56 | 0.578 |
| Time × Paternal Involvement | 0.02 | 0.04 | -0.05 – 0.10 | 0.63 | 0.528 |
| Random Effects | | | | | |
| σ^2^ | 0.27 | | | | |
| τ_00_ _ID_ | 0.00 | | | | |
| N _ID_ | 39 | | | | |
| Observations | 78 | | | | |
| Marginal R^2^ / Conditional R^2^ | 0.047 / NA | | | | |

*Note.* *Father Latency ~ Time + RF + Paternal Involvement + Time*RF + Time*Paternal Involvement + (1 | ID)*.

**Table S10**

*Mixed-Effects Model For Total Overlaps*

|  | Total Overlaps | | | | |
| --- | --- | --- | --- | --- | --- |
| *Predictors* | *Estimates* | *se* | *CI* | *Statistic* | *p* |
| (Intercept) | 13.74 | 4.21 | 5.34 – 22.15 | 3.26 | 0.002 |
| Time (T1) | -5.47 | 5.96 | -17.36 – 6.42 | -0.92 | 0.362 |
| RF | -0.75 | 0.71 | -2.17 – 0.67 | -1.05 | 0.295 |
| Paternal Involvement | -0.34 | 0.36 | -1.06 – 0.39 | -0.93 | 0.356 |
| Time × RF | 0.36 | 1.01 | -1.64 – 2.37 | 0.36 | 0.720 |
| Time × Paternal Involvement | 0.19 | 0.51 | -0.84 – 1.21 | 0.36 | 0.717 |
| Random Effects | | | | | |
| σ^2^ | 47.65 | | | | |
| τ_00_ _ID_ | 0.00 | | | | |
| N _ID_ | 39 | | | | |
| Observations | 78 | | | | |
| Marginal R^2^ / Conditional R^2^ | 0.066 / NA | | | | |

*Note.* *Overlaps ~ Time + RF + Paternal Involvement + Time*RF + Time*Paternal Involvement + (1 | ID)*.

**Table S11**

*Mixed-Effects Model For Infant Overlaps*

|  | Infant Overlaps | | | | |
| --- | --- | --- | --- | --- | --- |
| *Predictors* | *Estimates* | *se* | *CI* | *Statistic* | *p* |
| (Intercept) | 17.86 | 12.68 | -7.44 – 43.16 | 1.41 | 0.164 |
| Time (T1) | -0.53 | 17.94 | -36.31 – 35.24 | -0.03 | 0.976 |
| RF | 1.49 | 2.14 | -2.77 – 5.76 | 0.70 | 0.487 |
| Paternal Involvement | -0.01 | 1.10 | -2.20 – 2.17 | -0.01 | 0.990 |
| Time × RF | -1.77 | 3.03 | -7.80 – 4.27 | -0.58 | 0.561 |
| Time × Paternal Involvement | 0.91 | 1.55 | -2.18 – 4.00 | 0.59 | 0.557 |
| Random Effects | | | | | |
| σ^2^ | 431.67 | | | | |
| τ_00_ _ID_ | 0.00 | | | | |
| N _ID_ | 39 | | | | |
| Observations | 78 | | | | |
| Marginal R^2^ / Conditional R^2^ | 0.027 / NA | | | | |

*Note.* *Infant Overlaps ~ Time + RF + Paternal Involvement + Time*RF + Time*Paternal Involvement + (1 | ID)*.

**Table S12**

*Mixed-Effects Model For Father Overlaps*

|  | Father Overlaps | | | | |
| --- | --- | --- | --- | --- | --- |
| *Predictors* | *Estimates* | *se* | *CI* | *Statistic* | *p* |
| (Intercept) | 11.26 | 2.60 | 6.08 – 16.45 | 4.33 | <0.001 |
| Time (T1) | -6.00 | 3.35 | -12.69 – 0.68 | -1.79 | 0.077 |
| RF | -1.12 | 0.44 | -2.00 – -0.25 | -2.56 | 0.013 |
| Paternal Involvement | -0.24 | 0.22 | -0.68 – 0.21 | -1.05 | 0.298 |
| Time × RF | 0.63 | 0.57 | -0.49 – 1.76 | 1.12 | 0.265 |
| Time × Paternal Involvement | 0.14 | 0.29 | -0.43 – 0.72 | 0.49 | 0.623 |
| Random Effects | | | | | |
| σ^2^ | 15.05 | | | | |
| τ_00_ _ID_ | 3.08 | | | | |
| ICC | 0.17 | | | | |
| N _ID_ | 39 | | | | |
| Observations | 78 | | | | |
| Marginal R^2^ / Conditional R^2^ | 0.162 / 0.304 | | | | |

*Note.* *Father Overlaps ~ Time + RF + Paternal Involvement + Time*RF + Time*Paternal Involvement + (1 | ID).*

**Table S13**

*Mixed-Effects Model For Infant Overlap Duration*

|  | Infant Overlap Duration | | | | |
| --- | --- | --- | --- | --- | --- |
| *Predictors* | *Estimates* | *se* | *CI* | *Statistic* | *p* |
| (Intercept) | 0.21 | 0.14 | -0.07 – 0.49 | 1.47 | 0.146 |
| Time (T1) | 0.35 | 0.20 | -0.05 – 0.74 | 1.74 | 0.087 |
| RF | 0.05 | 0.02 | 0.00 – 0.09 | 2.00 | 0.050 |
| Paternal Involvement | 0.01 | 0.01 | -0.01 – 0.04 | 1.15 | 0.255 |
| Time × RF | -0.07 | 0.03 | -0.14 – -0.00 | -2.10 | 0.039 |
| Time × Paternal Involvement | -0.02 | 0.02 | -0.06 – 0.01 | -1.27 | 0.209 |
| Random Effects | | | | | |
| σ^2^ | 0.05 | | | | |
| τ_00_ _ID_ | 0.00 | | | | |
| N _ID_ | 39 | | | | |
| Observations | 78 | | | | |
| Marginal R^2^ / Conditional R^2^ | 0.081 / NA | | | | |

*Note.* *Infant Overlap Duration ~ Time + RF + Paternal Involvement + Time*RF + Time*Paternal Involvement + (1 | ID).*

**Table S14**

*Mixed-Effects Model For Father Overlap Duration*

|  | Father Overlap Duration | | | | |
| --- | --- | --- | --- | --- | --- |
| *Predictors* | *Estimates* | *std. Error* | *CI* | *Statistic* | *p* |
| (Intercept) | 0.76 | 0.23 | 0.30 – 1.22 | 3.31 | 0.001 |
| Time (T1) | -0.10 | 0.33 | -0.75 – 0.55 | -0.31 | 0.756 |
| RF | -0.00 | 0.04 | -0.08 – 0.08 | -0.03 | 0.979 |
| Paternal Involvement | -0.05 | 0.02 | -0.09 – -0.01 | -2.55 | 0.013 |
| Time × RF | -0.05 | 0.05 | -0.16 – 0.06 | -0.85 | 0.398 |
| Time × Paternal Involvement | 0.04 | 0.03 | -0.01 – 0.10 | 1.47 | 0.145 |
| Random Effects | | | | | |
| σ^2^ | 0.14 | | | | |
| τ_00_ _ID_ | 0.00 | | | | |
| N _ID_ | 39 | | | | |
| Observations | 78 | | | | |
| Marginal R^2^ / Conditional R^2^ | 0.121 / NA | | | | |

*Note.* *Father Overlap Duration ~ Time + RF + Paternal Involvement + Time*RF + Time*Paternal Involvement + (1 | ID)*.

| **Table S15**  *Mixed-Effects Model For Total Turn-Taking (with Covariates)* | | | | | |
| --- | --- | --- | --- | --- | --- |
|  | Turn-Taking | | | | |
| *Predictors* | *Estimates* | *std. Error* | *CI* | *Statistic* | *p* |
| (Intercept) | 39.87 | 12.29 | 15.33 – 64.40 | 3.24 | **0.002** |
| Time (T1) | 5.58 | 7.14 | -8.68 – 19.83 | 0.78 | 0.438 |
| RF | -0.59 | 1.17 | -2.92 – 1.74 | -0.51 | 0.615 |
| Paternal Involvement | -0.13 | 0.60 | -1.32 – 1.07 | -0.21 | 0.834 |
| Father Age | -0.54 | 0.28 | -1.09 – 0.01 | -1.96 | 0.054 |
| Infant Sex | -0.26 | 3.44 | -7.13 – 6.60 | -0.08 | 0.939 |
| Time × RF | -0.45 | 1.20 | -2.85 – 1.95 | -0.37 | 0.710 |
| Time × Paternal Involvement | -0.48 | 0.62 | -1.71 – 0.75 | -0.78 | 0.440 |
| **Random Effects** | | | | | |
| σ^2^ | 68.46 | | | | |
| τ_00_ _ID_ | 59.16 | | | | |
| ICC | 0.46 | | | | |
| N _ID_ | 39 | | | | |
| Observations | 78 | | | | |
| Marginal R^2^ / Conditional R^2^ | 0.086 / 0.510 | | | | |

*Note. Turn Taking ~ Time + RF + Paternal Involvement + Father Age + Infant Sex + Time*RF + Time*Paternal Involvement + (1 | ID)*.

| **Table S16**  *Mixed-Effects Model For Infant Turn-Taking (with Covariates)* | | | | | |
| --- | --- | --- | --- | --- | --- |
|  | Proportion of Turn-Taking FI | | | | |
| *Predictors* | *Estimates* | *std. Error* | *CI* | *Statistic* | *p* |
| (Intercept) | 38.15 | 26.65 | -15.04 – 91.34 | 1.43 | 0.157 |
| Time (T1) | 13.66 | 14.46 | -15.20 – 42.53 | 0.94 | 0.348 |
| RF | 2.75 | 2.50 | -2.23 – 7.73 | 1.10 | 0.275 |
| Paternal Involvement | 1.40 | 1.28 | -1.16 – 3.96 | 1.09 | 0.280 |
| Father Age | -0.70 | 0.60 | -1.90 – 0.51 | -1.15 | 0.253 |
| Infant Sex | 4.91 | 7.50 | -10.05 – 19.87 | 0.65 | 0.515 |
| Time × RF | -0.54 | 2.44 | -5.41 – 4.33 | -0.22 | 0.825 |
| Time × Paternal Involvement | -0.32 | 1.25 | -2.81 – 2.17 | -0.26 | 0.798 |
| **Random Effects** | | | | | |
| σ^2^ | 280.64 | | | | |
| τ_00_ _ID_ | 303.80 | | | | |
| ICC | 0.52 | | | | |
| N _ID_ | 39 | | | | |
| Observations | 78 | | | | |
| Marginal R^2^ / Conditional R^2^ | 0.115 / 0.575 | | | | |

*Note. Turn Taking FI ~ Time + RF + Paternal Involvement + Father Age + Infant Sex + Time*RF + Time*Paternal Involvement + (1 | ID).*

| **Table S17**  *Mixed-Effects Model For Father Turn-Taking (with Covariates)* | | | | | |
| --- | --- | --- | --- | --- | --- |
|  | Proportion of Turn-Taking IF | | | | |
| *Predictors* | *Estimates* | *std. Error* | *CI* | *Statistic* | *p* |
| (Intercept) | 34.17 | 9.77 | 14.67 – 53.67 | 3.50 | **0.001** |
| Time (T1) | -3.01 | 7.80 | -18.58 – 12.55 | -0.39 | 0.700 |
| RF | -1.30 | 1.01 | -3.30 – 0.71 | -1.29 | 0.202 |
| Paternal Involvement | -0.24 | 0.52 | -1.27 – 0.79 | -0.46 | 0.647 |
| Father Age | -0.39 | 0.21 | -0.81 – 0.04 | -1.83 | 0.072 |
| Infant Sex | -0.09 | 2.62 | -5.32 – 5.13 | -0.04 | 0.972 |
| Time × RF | 1.52 | 1.32 | -1.10 – 4.15 | 1.16 | 0.251 |
| Time × Paternal Involvement | -0.45 | 0.67 | -1.80 – 0.89 | -0.67 | 0.505 |
| **Random Effects** | | | | | |
| σ^2^ | 81.66 | | | | |
| τ_00_ _ID_ | 13.34 | | | | |
| ICC | 0.14 | | | | |
| N _ID_ | 39 | | | | |
| Observations | 78 | | | | |
| Marginal R^2^ / Conditional R^2^ | 0.093 / 0.220 | | | | |

*Note. Turn Taking IF ~ Time + RF + Paternal Involvement + Father Age + Infant Sex + Time*RF + Time*Paternal Involvement + (1 | ID).*

| **Table S18**  *Mixed-Effects Model For Total Turn-Taking Latency (with Covariates)* | | | | | |
| --- | --- | --- | --- | --- | --- |
|  | Latency | | | | |
| *Predictors* | *Estimates* | *std. Error* | *CI* | *Statistic* | *p* |
| (Intercept) | 0.80 | 0.31 | 0.18 – 1.42 | 2.56 | **0.013** |
| Time (T1) | 0.32 | 0.28 | -0.24 – 0.87 | 1.13 | 0.263 |
| RF | -0.00 | 0.03 | -0.07 – 0.07 | -0.00 | 0.999 |
| Paternal Involvement | -0.02 | 0.02 | -0.05 – 0.02 | -0.92 | 0.361 |
| Father Age | 0.01 | 0.01 | -0.01 – 0.02 | 0.79 | 0.431 |
| Infant Sex | -0.09 | 0.08 | -0.25 – 0.07 | -1.08 | 0.282 |
| Time × RF | -0.07 | 0.05 | -0.16 – 0.02 | -1.49 | 0.141 |
| Time × Paternal Involvement | 0.01 | 0.02 | -0.04 – 0.06 | 0.47 | 0.640 |
| **Random Effects** | | | | | |
| σ^2^ | 0.10 | | | | |
| τ_00_ _ID_ | 0.00 | | | | |
| N _ID_ | 39 | | | | |
| Observations | 78 | | | | |
| Marginal R^2^ / Conditional R^2^ | 0.106 / NA | | | | |

*Note. Latency ~ Time + RF + Paternal Involvement + Father Age + Infant Sex + Time*RF + Time*Paternal Involvement + (1 | ID).*

| **Table S19**  *Mixed-Effects Model For Infant Turn-Taking Latency (with Covariates)* | | | | | |
| --- | --- | --- | --- | --- | --- |
|  | Latency Infant | | | | |
| *Predictors* | *Estimates* | *std. Error* | *CI* | *Statistic* | *p* |
| (Intercept) | 0.84 | 0.40 | 0.05 – 1.63 | 2.12 | **0.038** |
| Time (T1) | 0.26 | 0.34 | -0.43 – 0.95 | 0.75 | 0.453 |
| RF | -0.02 | 0.04 | -0.10 – 0.06 | -0.46 | 0.647 |
| Paternal Involvement | -0.01 | 0.02 | -0.06 – 0.03 | -0.65 | 0.520 |
| Father Age | 0.00 | 0.01 | -0.01 – 0.02 | 0.26 | 0.794 |
| Infant Sex | 0.12 | 0.10 | -0.08 – 0.33 | 1.18 | 0.241 |
| Time × RF | -0.05 | 0.06 | -0.17 – 0.06 | -0.92 | 0.363 |
| Time × Paternal Involvement | 0.01 | 0.03 | -0.05 – 0.07 | 0.21 | 0.836 |
| **Random Effects** | | | | | |
| σ^2^ | 0.16 | | | | |
| τ_00_ _ID_ | 0.01 | | | | |
| ICC | 0.04 | | | | |
| N _ID_ | 39 | | | | |
| Observations | 78 | | | | |
| Marginal R^2^ / Conditional R^2^ | 0.063 / 0.100 | | | | |

*Note. Latency Infant ~ Time + RF + Paternal Involvement + Father Age + Infant Sex + Time*RF + Time*Paternal Involvement + (1 | ID).*

| **Table S20**  *Mixed-Effects Model For Father Turn-Taking Latency (with Covariates)* | | | | | |
| --- | --- | --- | --- | --- | --- |
|  | Latency Father | | | | |
| *Predictors* | *Estimates* | *std. Error* | *CI* | *Statistic* | *p* |
| (Intercept) | 0.75 | 0.50 | -0.25 – 1.76 | 1.49 | 0.140 |
| Time (T1) | 0.19 | 0.45 | -0.71 – 1.10 | 0.43 | 0.668 |
| RF | 0.03 | 0.05 | -0.08 – 0.14 | 0.54 | 0.593 |
| Paternal Involvement | -0.03 | 0.03 | -0.09 – 0.02 | -1.15 | 0.256 |
| Father Age | 0.00 | 0.01 | -0.02 – 0.03 | 0.41 | 0.687 |
| Infant Sex | -0.11 | 0.13 | -0.37 – 0.16 | -0.81 | 0.423 |
| Time × RF | -0.04 | 0.08 | -0.19 – 0.11 | -0.55 | 0.581 |
| Time × Paternal Involvement | 0.02 | 0.04 | -0.05 – 0.10 | 0.63 | 0.531 |
| **Random Effects** | | | | | |
| σ^2^ | 0.27 | | | | |
| τ_00_ _ID_ | 0.00 | | | | |
| N _ID_ | 39 | | | | |
| Observations | 78 | | | | |
| Marginal R^2^ / Conditional R^2^ | 0.058 / NA | | | | |

*Note. Latency Father ~ Time + RF + Paternal Involvement + Father Age + Infant Sex + Time*RF + Time*Paternal Involvement + (1 | ID).*

| **Table S21**  *Mixed-Effects Model For Total Overlaps (with Covariates)* | | | | | |
| --- | --- | --- | --- | --- | --- |
|  | Overlap | | | | |
| *Predictors* | *Estimates* | *std. Error* | *CI* | *Statistic* | *p* |
| (Intercept) | 5.02 | 6.58 | -8.12 – 18.15 | 0.76 | 0.448 |
| Time (T1) | -5.47 | 5.90 | -17.24 – 6.30 | -0.93 | 0.357 |
| RF | -0.71 | 0.70 | -2.12 – 0.69 | -1.01 | 0.315 |
| Paternal Involvement | -0.28 | 0.36 | -1.00 – 0.44 | -0.77 | 0.444 |
| Father Age | 0.25 | 0.14 | -0.02 – 0.53 | 1.82 | 0.073 |
| Infant Sex | 0.00 | 1.72 | -3.43 – 3.44 | 0.00 | 0.998 |
| Time × RF | 0.36 | 1.00 | -1.62 – 2.35 | 0.36 | 0.717 |
| Time × Paternal Involvement | 0.19 | 0.51 | -0.83 – 1.20 | 0.37 | 0.714 |
| **Random Effects** | | | | | |
| σ^2^ | 46.70 | | | | |
| τ_00_ _ID_ | 0.00 | | | | |
| N _ID_ | 39 | | | | |
| Observations | 78 | | | | |
| Marginal R^2^ / Conditional R^2^ | 0.105 / NA | | | | |

*Note. Overlap ~ Time + RF + Paternal Involvement + Father Age + Infant Sex + Time*RF + Time*Paternal Involvement + (1 | ID).*

| **Table S22**  *Mixed-Effects Model For Infant Overlaps (with Covariates)* | | | | | |
| --- | --- | --- | --- | --- | --- |
|  | Overlap Infant | | | | |
| *Predictors* | *Estimates* | *std. Error* | *CI* | *Statistic* | *p* |
| (Intercept) | -15.97 | 19.58 | -55.03 – 23.10 | -0.82 | 0.418 |
| Time (T1) | -0.53 | 17.55 | -35.56 – 34.49 | -0.03 | 0.976 |
| RF | 1.46 | 2.10 | -2.72 – 5.65 | 0.70 | 0.487 |
| Paternal Involvement | 0.17 | 1.08 | -1.98 – 2.32 | 0.16 | 0.876 |
| Father Age | 0.87 | 0.41 | 0.05 – 1.69 | 2.11 | **0.039** |
| Infant Sex | 6.57 | 5.12 | -3.64 – 16.78 | 1.28 | 0.203 |
| Time × RF | -1.77 | 2.96 | -7.68 – 4.14 | -0.60 | 0.552 |
| Time × Paternal Involvement | 0.91 | 1.52 | -2.11 – 3.94 | 0.60 | 0.549 |
| **Random Effects** | | | | | |
| σ^2^ | 413.34 | | | | |
| τ_00_ _ID_ | 0.00 | | | | |
| N _ID_ | 39 | | | | |
| Observations | 78 | | | | |
| Marginal R^2^ / Conditional R^2^ | 0.088 / NA | | | | |

*Note. Overlap Infant ~ Time + RF + Paternal Involvement + Father Age + Infant Sex + Time*RF + Time*Paternal Involvement + (1 | ID).*

| **Table S23**  *Mixed-Effects Model For Father Overlaps (with Covariates)* | | | | | |
| --- | --- | --- | --- | --- | --- |
|  | Overlap Father | | | | |
| *Predictors* | *Estimates* | *std. Error* | *CI* | *Statistic* | *p* |
| (Intercept) | 6.38 | 4.29 | -2.19 – 14.95 | 1.49 | 0.142 |
| Time (T1) | -6.00 | 3.35 | -12.69 – 0.68 | -1.79 | 0.077 |
| RF | -1.13 | 0.44 | -2.00 – -0.25 | -2.57 | **0.012** |
| Paternal Involvement | -0.21 | 0.23 | -0.66 – 0.24 | -0.93 | 0.356 |
| Father Age | 0.12 | 0.09 | -0.06 – 0.31 | 1.33 | 0.187 |
| Infant Sex | 1.03 | 1.16 | -1.27 – 3.34 | 0.89 | 0.375 |
| Time × RF | 0.63 | 0.57 | -0.49 – 1.76 | 1.12 | 0.266 |
| Time × Paternal Involvement | 0.14 | 0.29 | -0.43 – 0.72 | 0.49 | 0.623 |
| Random Effects | | | | | |
| σ^2^ | 15.05 | | | | |
| τ_00_ _ID_ | 3.03 | | | | |
| ICC | 0.17 | | | | |
| N _ID_ | 39 | | | | |
| Observations | 78 | | | | |
| Marginal R^2^ / Conditional R^2^ | 0.185 / 0.321 | | | | |

*Note. Overlap Father ~ Time + RF + Paternal Involvement + Father Age + Infant Sex + Time*RF + Time*Paternal Involvement + (1 | ID).*

| **Table S24**  *Mixed-Effects Model For Infant Overlap Duration (with Covariates)* | | | | | |
| --- | --- | --- | --- | --- | --- |
|  | Overlap duration infant | | | | |
| *Predictors* | *Estimates* | *std. Error* | *CI* | *Statistic* | *p* |
| (Intercept) | -0.17 | 0.21 | -0.60 – 0.25 | -0.81 | 0.419 |
| Time (T1) | 0.35 | 0.19 | -0.04 – 0.73 | 1.81 | 0.075 |
| RF | 0.05 | 0.02 | 0.00 – 0.10 | 2.18 | **0.032** |
| Paternal Involvement | 0.02 | 0.01 | -0.01 – 0.04 | 1.43 | 0.157 |
| Father Age | 0.01 | 0.00 | 0.00 – 0.02 | 2.57 | **0.012** |
| Infant Sex | -0.03 | 0.06 | -0.14 – 0.08 | -0.60 | 0.549 |
| Time × RF | -0.07 | 0.03 | -0.13 – -0.01 | -2.19 | **0.032** |
| Time × Paternal Involvement | -0.02 | 0.02 | -0.05 – 0.01 | -1.32 | 0.191 |
| **Random Effects** | | | | | |
| σ^2^ | 0.05 | | | | |
| τ_00_ _ID_ | 0.00 | | | | |
| N _ID_ | 39 | | | | |
| Observations | 78 | | | | |
| Marginal R^2^ / Conditional R^2^ | 0.166 / NA | | | | |

*Note. Overlap duration infant ~ Time + RF + Paternal Involvement + Father Age + Infant Sex + Time*RF + Time*Paternal Involvement + (1 | ID).*

| **Table S25**  *Mixed-Effects Model For Father Overlap Duration (with Covariates)* | | | | | |
| --- | --- | --- | --- | --- | --- |
|  | Overlap duration father | | | | |
| *Predictors* | *Estimates* | *std. Error* | *CI* | *Statistic* | *p* |
| (Intercept) | 0.59 | 0.37 | -0.15 – 1.32 | 1.59 | 0.115 |
| Time (T1) | -0.10 | 0.33 | -0.76 – 0.56 | -0.31 | 0.759 |
| RF | -0.00 | 0.04 | -0.08 – 0.08 | -0.03 | 0.978 |
| Paternal Involvement | -0.05 | 0.02 | -0.09 – -0.01 | -2.46 | **0.016** |
| Father Age | 0.00 | 0.01 | -0.01 – 0.02 | 0.59 | 0.554 |
| Infant Sex | 0.03 | 0.10 | -0.16 – 0.22 | 0.32 | 0.753 |
| Time × RF | -0.05 | 0.06 | -0.16 – 0.06 | -0.84 | 0.404 |
| Time × Paternal Involvement | 0.04 | 0.03 | -0.02 – 0.10 | 1.46 | 0.150 |
| **Random Effects** | | | | | |
| σ^2^ | 0.15 | | | | |
| τ_00_ _ID_ | 0.00 | | | | |
| N _ID_ | 39 | | | | |
| Observations | 78 | | | | |
| Marginal R^2^ / Conditional R^2^ | 0.122 / NA | | | | |

*Note. Overlap duration father ~ Time + RF + Paternal Involvement + Father Age + Infant Sex + Time*RF + Time*Paternal Involvement + (1 | ID).*

| **Table S26**  *Mixed-Effects Models Predicting Turn-Taking (1s Criterion)* | | | | | | | | | | | | | | | |
| --- | --- | --- | --- | --- | --- | --- | --- | --- | --- | --- | --- | --- | --- | --- | --- |
|  | **Total Turn-Taking (1s)** | | | | | **Infant Turns (1s)** | | | | | **Father Turns (1s)** | | | | |
| *Predictors* | *Estimates* | *SE* | *CI* | *Statistic* | *p* | *Estimates* | *SE* | *CI* | *Statistic* | *p* | *Estimates* | *SE* | *CI* | *Statistic* | *p* |
| (Intercept) | 22.66 | 12.57 | -2.42 – 47.73 | 1.80 | 0.076 | 10.12 | 10.60 | -11.03 – 31.27 | 0.95 | 0.343 | 12.54 | 4.73 | 3.10 – 21.97 | 2.65 | **0.010** |
| Time | 2.02 | 14.86 | -27.63 – 31.66 | 0.14 | 0.892 | -1.21 | 13.14 | -27.41 – 24.99 | -0.09 | 0.927 | 3.23 | 6.11 | -8.95 – 15.40 | 0.53 | 0.599 |
| RF | 1.18 | 2.12 | -3.05 – 5.41 | 0.56 | 0.580 | 2.13 | 1.79 | -1.44 – 5.70 | 1.19 | 0.238 | -0.95 | 0.80 | -2.54 – 0.64 | -1.19 | 0.239 |
| Paternal Involvement | 1.16 | 1.09 | -1.01 – 3.32 | 1.06 | 0.291 | 0.87 | 0.92 | -0.96 – 2.70 | 0.95 | 0.347 | 0.29 | 0.41 | -0.53 – 1.10 | 0.70 | 0.484 |
| Time × RF | 2.69 | 2.51 | -2.31 – 7.69 | 1.07 | 0.287 | 2.60 | 2.22 | -1.82 – 7.01 | 1.17 | 0.245 | 0.09 | 1.03 | -1.96 – 2.15 | 0.09 | 0.927 |
| Time × Paternal Involvement | -1.02 | 1.28 | -3.58 – 1.54 | -0.79 | 0.431 | -0.61 | 1.13 | -2.87 – 1.65 | -0.54 | 0.592 | -0.41 | 0.53 | -1.46 – 0.65 | -0.77 | 0.445 |
| **Random Effects** | | | | | | | | | | | | | | | |
| σ^2^ | 296.39 | | | | | 231.46 | | | | | 50.00 | | | | |
| τ_00_ | 127.76 _ID_ | | | | | 70.26 _ID_ | | | | | 10.04 _ID_ | | | | |
| ICC | 0.30 | | | | | 0.23 | | | | | 0.17 | | | | |
| N | 39 _ID_ | | | | | 39 _ID_ | | | | | 39 _ID_ | | | | |
| Observations | 78 | | | | | 78 | | | | | 78 | | | | |
| Marginal R^2^ / Conditional R^2^ | 0.099 / 0.371 | | | | | 0.141 / 0.341 | | | | | 0.061 / 0.218 | | | | |

| **Table S27**  *Mixed-Effects Models Predicting Turn-Taking (2s Criterion)* | | | | | | | | | | | | | | | |
| --- | --- | --- | --- | --- | --- | --- | --- | --- | --- | --- | --- | --- | --- | --- | --- |
|  | **Total Turn-Taking (2s)** | | | | | **Infant Turns (2s)** | | | | | **Father Turns (2s)** | | | | |
| *Predictors* | *Estimates* | *SE* | *CI* | *Statistic* | *p* | *Estimates* | *SE* | *CI* | *Statistic* | *p* | *Estimates* | *SE* | *CI* | *Statistic* | *p* |
| (Intercept) | 30.85 | 16.39 | -1.84 – 63.54 | 1.88 | 0.064 | 13.80 | 14.18 | -14.50 – 42.09 | 0.97 | 0.334 | 17.06 | 6.82 | 3.46 – 30.65 | 2.50 | **0.015** |
| Time | 19.13 | 18.96 | -18.69 – 56.95 | 1.01 | 0.317 | 5.87 | 16.75 | -27.54 – 39.29 | 0.35 | 0.727 | 13.26 | 8.61 | -3.91 – 30.42 | 1.54 | 0.128 |
| RF | 2.23 | 2.76 | -3.29 – 7.74 | 0.81 | 0.423 | 3.29 | 2.39 | -1.49 – 8.06 | 1.37 | 0.174 | -1.06 | 1.15 | -3.35 – 1.23 | -0.92 | 0.360 |
| Paternal Involvement | 1.38 | 1.42 | -1.45 – 4.20 | 0.97 | 0.334 | 1.30 | 1.23 | -1.14 – 3.75 | 1.06 | 0.292 | 0.08 | 0.59 | -1.10 – 1.25 | 0.13 | 0.899 |
| Time × RF | 0.33 | 3.20 | -6.05 – 6.71 | 0.10 | 0.918 | 1.60 | 2.83 | -4.03 – 7.24 | 0.57 | 0.573 | -1.27 | 1.45 | -4.17 – 1.62 | -0.88 | 0.383 |
| Time × Paternal Involvement | -2.22 | 1.64 | -5.49 – 1.05 | -1.35 | 0.180 | -1.47 | 1.45 | -4.35 – 1.42 | -1.01 | 0.314 | -0.75 | 0.74 | -2.23 – 0.73 | -1.01 | 0.316 |
| **Random Effects** | | | | | | | | | | | | | | | |
| σ^2^ | 482.39 | | | | | 376.53 | | | | | 99.36 | | | | |
| τ_00_ | 238.25 _ID_ | | | | | 163.28 _ID_ | | | | | 25.34 _ID_ | | | | |
| ICC | 0.33 | | | | | 0.30 | | | | | 0.20 | | | | |
| N | 39 _ID_ | | | | | 39 _ID_ | | | | | 39 _ID_ | | | | |
| Observations | 78 | | | | | 78 | | | | | 78 | | | | |
| Marginal R^2^ / Conditional R^2^ | 0.069 / 0.377 | | | | | 0.100 / 0.372 | | | | | 0.095 / 0.279 | | | | |
